# Modifiable Factors and Colon Cancer Risk in Thai Population

**DOI:** 10.31557/APJCP.2021.22.1.37

**Published:** 2021-01

**Authors:** Suthat Chottanapund, Kanittha Chamroonsawasdi, Pravich Tunyasitthisundhorn, Wichai Aekplakorn, Pimpan Silpasuwan, Puree Anantachoti, Nipa Rojroongwasinkul, Sanga Damapong, Bundit Sornpaisarn, Wiwat Rojanapithayakorn, Kumnuan Ungchusak

**Affiliations:** 1 *Bamrasnaradura Infectious Disease Institute, Ministry of Public Health, Nontaburi 11000, Thailand. *; 2 *Department of Family Health, Faculty of Public Health, Mahidol University, Bangkok 10400, Thailand. *; 3 *Medical Research Network of the Consortium of Thai Medical Schools, Bangkok 10900, Thailand. *; 4 *Faculty of Medicine, Ramathibodi Hospital, Bangkok 10400, Thailand. *; 5 *Faculty of Public Health, Mahidol University, Bangkok 10400, Thailand.*; 6 *Department of Social and Administrative Pharmacy, Faculty of Pharmaceutical Sciences, Chulalongkorn University, Bangkok 10330 Thailand. *; 7 *Institute of Nutrition, Mahidol University, Nakhon Pathom 73170, Thailand. *; 8 *Bureau of Health Promotion, Department of Health, Ministry of Public Health, Nonthaburi, 11000, Thailand. *; 9 *Thai Health Promotion Foundation, Bangkok 10120, Thailand. *; 10 *Bureau of Epidemiology, Nonthaburi 11000, Thailand. *

**Keywords:** Ca colon, physical activity, smoking, non-communicable diseases, risk

## Abstract

To demonstrate the possible impact of modifiable factors on colon cancer development in Thai population, we conducted this case-control study from June 2016 until June 2017. The study was conducted in 11 Thai provincial hospitals. The hospitals in this study were selected by stratification by regions. Patients included 504 ones who were newly diagnosed with colon cancer within 1 month. In the control group, 997 health individuals were enrolled. Both case and control were adjusted by age. The results of this study showed that age and socioeconomic factors were associated with colon cancer risk. In addition, it was found that family history of colon cancer had very high association with colon cancer risk. Behavioral factors, including smoking, inadequate physical exercise, and salty food consumption were associated with colon cancer. We detected no association between obesity, alcohol consumption, and colon cancer. The results suggested that colon cancer might have higher association with genetic factors than behavioral factors among Thai patients. Based on the results of this study, stop smoking and promote adequate physical activity are suggested to reduce the incidence of colon cancrr among Thai patients.

## Introduction

World Health Organization (WHO) defines non-communicable diseases (NCDs) as chronic diseases that are not communicable between people. There are four common types of NCDs, namely cardiovascular diseases, cancers, chronic respiratory diseases (COPD), and diabetes mellitus (DM) (Kessaram et al., 2015; WHO, 2018). Colonic cancer is the third common cause of death in Thai population, especially in male patients (WHO, 2017, 2018). Colonic cancer is caused by the abnormal growth of colonic epithelial cells, resulting in an aggressive tumor (Faivre, 1994). Most of sufferers complained of symptoms of intestinal compression (Faivre, 1994). According to its nature, colonic cancer can be divided into 3 types, namely sporadic colonic cancer (over 70 % ), familial colonic cancer (approximate 25 %), and hereditary colonic cancer (approximate 5 %) (Faivre, 1994). Nowadays, multimodality of treatments can prolong survival of the patients; nevertheless, the mortality and morbidity of the disease are high. Moreover, colonic cancer treatment costs become higher than in the past due to development of new-targeted therapies (Faivre, 1994; Lerdkiattikorn et al., 2015). Prevention can be one of the most cost-effective measures for low resources countries such as Thailand (Khuhaprema and Srivatanakul, 2008; Lepage et al., 2010). 

To prevent all NCDs, WHO and United Nations proposed a prevention and control model for NCDs called a 4 × 4 model (WHO, 2018). The 4 × 4 model comprises of four risk behaviors, namely tobacco use, physical inactivity, unhealthy diet, and the harmful use of alcohol and four abnormal physiology markers, including hyperglycemia, hyperlipidemia, hypertension, and overweight as well as obesity (Demirbas, 2015; Slattery et al., 1990; WHO, 2017, 2018; Wolin et al., 2009). For meta-analyses found that many inflammatory bowel diseases and heredity factor had the highest association with development of colon cancer (Bye et al., 2017). For modifiable risks are obesity, smoking, exercise, and eating red meat (Khuhaprema and Srivatanakul, 2008; Wheat et al., 2016). To demonstrate the impact of modifiable risks on colon cancer in Thai population, we developed a survey questionnaire comprising of three parts, namely subject profile, standard questionnaire for smoking behaviors; excessive alcohol consumption and physical exercise; and cognitive-behavioral factors. The questionnaire was tested and validated in Samuprakarn province, Thailand. Our study aimed to show the odd ratio of modifiable risks of colonic cancer, including tobacco use, physical inactivity, and unhealthy diet in Thai population according to 4 x 4 model proposed by WHO. 

## Materials and Methods

We conducted this case-control study from June 2016 until June 2017. The study was done in 11 Thai provincial hospitals. The hospitals in this study were stratifically selected by regions. The sample size was calculated using odd ratio. For this purpose, we set the significant odd ratio at 1.5 (n=990 per group). The formula for odd ratio was as follows:

pcase exp=ORPcontrexpol pcontrol expOR-1+1

We enrolled all newly diagnosed colon cancer within 1 month from outpatient clinics and hospital wards (n=504). For control group, we recruited individuals who came to the hospital for health check up. They were all healthy (n=997). Inclusion criteria were being Thai and age above 35 years old. Exclusion criteria were being non-Thai and critically ill. Case record form was developed from standard NCDs questionnaires, namely Cage test for cigarette smoking (Williams, 2014), Fagerstrom for nicotine dependence (Ebbert et al., 2006; Uysal et al., 2004), AUDIT for alcoholic dependence (Conigraveet al., 1995; Nordqvist et al., 2004), and WHO STEP wise for physical activity (WHO, 2017). The questionnaire was tested for reliability and validity in Samut Prakarn province, Thailand (Chamroonsawasdi et al., 2017). Content validity of the questionnaire was determined by the mean of the item content validity index (I-CVI) from five experts. The mean I-CVI ranged from 0.90–1.00 and for difficulty of knowledge ranged from 0.3–0.9. The reliability test of knowledge by KR-20 ranged from 0.648–0.799. The data were analyzed using t-test, Chi-square, and multiple logistic regression analysis. P-value < 0.05 was set as statistically significant.

All patients who enrolled in the study completed a written informed consent. In addition, these patients received full information and explanation about the study objectives and rights that a patient had before enrolling in the study. The study was approved by the Ethics Committee of each hospital (Maharaj Nakorn Si Thammarat Hospital EC. no. 39/2559, Surin Hospital EC. no.11/2559, REC-Hat Yai no. RYH 11/2559, Ayuttaya Hospital EC. no. 17/2559, Rayong Hospital EC. no. 11/2559, Lumpang hospital EC. no.64/2559, Udontani Hospital EC no. 32.102/1090, Pharhol Phonphayuhasana Hospital EC. No. 2016-06, Prathumtani Hospital EC. No. 32.203.3/1962, RBHEC 23/59, and Buddhachinaraj Hospital EC. No.6/60). This research was carried out in accordance with the Code of Ethics of the World Medical Association (Declaration of Helsinki). 

**Table 1:** Baseline Demographic Characteristics of the Participants

Demographic data		Case N (%) n =504	Control N (%) n = 997	p-value
Age	> 60	24 (4.8)	7 (0.7)	<0.001
	40-59	228 (45.4)	99 (9.9)	<0.001
	35-39	250 (49.8)	891 (89.4)	
Sex	Male	285 (56.8)	300 (30.1)	<0.001
	Female	217 (43.2)	697 (69.9)	
Occupation	Unemploy or housewife	189 (39.4)	79 (8.3)	<0.001
	Labour	69 (14.4)	122 (12.8)	<0.001
	Farmer	138 (28.8)	74 (7.7)	<0.001
	Private company	6 (1.3)	122 (12.8)	0.465
	Business owner	44 (9.2)	63 (6.6)	<0.001
	Government officer	34 (7.1)	496 (51.9)	
Education	Primary school	347 (69.3)	229 (23.0)	<0.001
	Secondary school	48 (9.6)	110 (11.1)	<0.001
	High school	72 (14.4)	272 (27.4)	<0.001
	Bachelor or higher	34 (6.8)	383 (38.5)	
Marital status	Married	365 (73.0)	670 (67.6)	0.033
	Single or divorce	135 (27.0)	321 (32.4)	
Health payment	30 bath scheme	379 (76.6)	253 (25.5)	<0.001
	Social insurance	43 (8.7)	351 (35.3)	0.039
	Civil servant	73 (14.7)	390 (39.2)	
Incomes	< 10,000	175 (67.0)	163 (31.7)	<0.001
	10,000 - 19,999	43 (16.5)	129 (25.1)	0.025
	> 20,000	43 (16.5)	222 (43.2)	
Family incomes	< 10,000	114 (46.5)	46 (10.8)	<0.001
	10,000 - 19,999	61 (24.9)	83 (19.4)	<0.001
	> 20,000	70 (28.6)	298 (69.8)	
Family history of colon cancer	No	3 (0.6)	943 (94.6)	
	Yes	480 (95.6)	65 (6.5)	<0.001

**Table 2:** The association between Physiological Factors and Colon Cancer Risk Based on Univaiate Analysis

Physiological factor	Case N (%) n =504	Control N (%) n = 997	p-value
BMI (km/m^2^)			
> 30.0 (Obese)	22 (4.4)	58 (5.9)	<0.01
25.0-29.9 (Overweight)	84 (16.9)	246 (25.0)	
18.5-24.9 (Normal)	295 (59.5)	646 (65.7)	
< 18.5 (Underweight)	95 (19.2)	33 (3.4)	
Systolic blood pressure (mmHg)			
> 160 (Stage II)	14 (2.8)	3 (0.4)	<0.01
140-159 (Stage I)	75 (15.2)	64 (8.0)	
121-139 (Pre hypertension)	163 (33.0)	257 (32.1)	
< 120 (Normal)	242 (49.0)	476 (59.5)	
Diastolic blood pressure (mmHg)			
> 90 (Hypertension)	40 (8.1)	57 (7.1)	0.59
80-89 (Pre-hypertension)	112 (22.6)	198 (24.8)	
< 80 (Normal)	343 (69.3)	544 (68.1)	
FBS (mg/dL)			
> 126 (Abnormal)	40 (35.1)	3 (0.5)	<0.01
< 126 (Normal)	74 (64.9)	580 (99.5)	
Physiological factor	Case N (%) n =504	Control N (%) n = 997	p-value
Cholesterol (mg/dL)			
> 240 (High)	15 (16.1)	74 (12.4)	0.3
200-239 (Borderline)	23 (24.7)	190 (31.9)	
< 200 (Normal)	55 (59.1)	331 (55.6)	
LDL (mg/dL)			
> 160 (High)	12 (17.6)	56 (14.0)	0.07
130-159 (Borderline)	10 (14.7)	111 (27.8)	
< 130 (Normal)	46 (67.6)	233 (58.3)	
HDL (mg/dL)			
< 40 (Abnormal)	22 (34.4)	32 (7.9)	<0.01
40-59	34 (53.1)	213 (52.3)	
> 60 (Normal)	8 (12.5)	162 (39.8)	
Triglyceride (mg/dL)			
> 150 (Abnormal)	24 (34.8)	93 (15.8)	<0.01
< 150 (Normal)	45 (65.2)	497 (84.2)	

**Table 3:** Association between Physiological Factors and Colon Cancer Risk

Physiological factor	Cases N (%)	Control N (%)	Univariate Logistic Adjusted Age, Gender, Occupation		Multivariate Logistic	
			OR (95% CI)	p	OR (95% CI)	p
BMI (km/m^2^)						
> 30.0 (Obese)	22 (4.4)	58 (5.9)	0.77 (0.39-1.52)	0.44	0.25 (0.05-1.30)	0.1
25.0-29.9 (Overweight)	84 (16.9)	246 (25.0)	0.88 (0.61-1.26)	0.47	0.83 (0.34-2.03)	0.69
18.5-24.9 (Normal)	295 (59.5)	646 (65.7)	1		1	
< 18.5 (Underweight)	95 (19.2)	33 (3.4)	6.2 (3.56-10.81)	<0.01	5.26 (1.10-25.14)	0.04
Systolic blood pressure (mmHg)						
> 160 (Stage II)	14 (2.8)	3 (0.4)	5.2 (1.02-26.56)	0.048	30.48 (1.85-502.8)	0.02
140-159 (Stage I)	75 (15.2)	64 (8.0)	0.83 (0.51-1.35)	0.45	0.76 (0.20-2.84)	0.69
121-139 (Pre hypertension)	163 (33.0)	257 (32.1)	0.7 (0.50-1.00)	0.049	0.89 (0.38-2.07)	0.79
< 120 (Normal)	242 (49.0)	476 (59.5)	1		1	
Diastolic blood pressure (mmHg)						
> 90 (Hypertension)	40 (8.1)	57 (7.1)	0.86 (0.50-1.49)	0.59		
80-89 (Pre-hypertension)	112 (22.6)	198 (24.8)	0.78 (0.54-1.13)	0.18		
< 80 (Normal)	343 (69.3)	544 (68.1)	1			
FBS (mg/dL)						
> 126 (Abnormal)	40 (35.1)	3 (0.5)	116.06 (25.20-534.58)	<0.001		
< 126 (Normal)	74 (64.9)	580 (99.5)	1			
Cholesterol (mg/dL)						
> 240 (High)	15 (16.1)	74 (12.4)	1.43 (0.63-3.26)	0.39		
200-239 (Borderline)	23 (24.7)	190 (31.9)	1.24 (0.60-2.57)	0.56		
< 200 (Normal)	55 (59.1)	331 (55.6)	1			
LDL (mg/dL)						
> 160 (High)	12 (17.6)	56 (14.0)	1.44 (0.60-3.45)	0.42		
130-159 (Borderline)	10 (14.7)	111 (27.8)	0.9 (0.37-2.22)	0.82		
< 130 (Normal)	46 (67.6)	233 (58.3)	1			
HDL (mg/dL)						
< 40 (Abnormal)	22 (34.4)	32 (7.9)	7.16 (2.34-21.94)	0.001	8.8 (2.62-29.5)	<0.001
40-59	34 (53.1)	213 (52.3)	1.76 (0.67-4.64)	0.25	1.88 (0.65-5.40)	0.24
> 60 (Normal)	8 (12.5)	162 (39.8)	1		1	
Triglyceride (mg/dL)						
> 150 (Abnormal)	24 (34.8)	93 (15.8)	1.55 (0.74-3.23)	0.24		
< 150 (Normal)	45 (65.2)	497 (84.2)	1			

**Table 4:** Association between Behavior Factors and Colon Cancer Risk

Behavioral factors	Case N (%) n =504	Control N (%) n = 997	p-value
Alcoholic drinking			
Dependent (>20)	9 (1.8)	17 (1.7)	0.24
Harmful (16-19)	4 (0.8)	19 (1.9)	
Hazardous (<16)	25 (5.0)	65 (6.5)	
Low Risk/ Non-drink	462 (92.4)	895 (89.9)	
Smoking (Pack-years)			
>20	33 (6.6)	9 (0.9)	<0.01
<20	92 (18.4)	84 (8.4)	
Non-smoker	374 (74.9)	902 (90.7)	
Cage score (Cigarette)			
Dependent (Score 1-4)	28 (2800.0)	64 (914.3)	0.28
Not dependent	1 (100.0)	7 (100.0)	
FTND score			
Score > 6 (High)	3 (0.6)	2 (0.2)	<0.01
Score < 6 (Low)	26 (5.2)	69 (6.9)	
Ex-smoker	98 (19.6)	23 (2.3)	
Non-smoker	374 (74.7)	902 (90.6)	
Physical activity			
Inadequate	452 (90.0)	860 (86.3)	0.04
Adequate	50 (10.0)	137 (13.7)	
Sweet food			
Frequently	242 (48.2)	567 (57.0)	<0.01
Rarely	260 (51.8)	428 (43.0)	
Fat food			
Frequently	333 (66.9)	643 (64.9)	<0.01
Rarely	165 (33.1)	347 (35.1)	
Salty food			
Frequently	298 (59.4)	502 (50.4)	<0.01
Rarely	204 (40.6)	495 (49.6)	

**Table 5:** Association Between Behavioral Factors and Colon Cancer Risk

Behavioral factors	Case N (%)	Control N (%)	Univariate Logistic Adjusted Age, Gender, Occupation		Multivariate Logistic	
			OR (95% CI)	p	OR (95% CI)	p
Alcoholic drinking						
Dependent (>20)	9 (1.8)	17 (1.7)	0.78 (0.27-2.25)	0.65		
Harmful (16-19)	4 (0.8)	19 (1.9)	0.34 (0.09-1.37)	0.13		
Hazardous (<16)	25 (5.0)	65 (6.5)	0.56 (0.31-1.04)	0.07		
Low Risk/ Non-drink	462 (92.4)	895 (89.9)	1			
Smoking (Pack-years)						
>20	33 (6.6)	9 (0.9)	3.00 (1.19-7.55)	0.02	3.25 (1.17-9.03)	0.024
<20	92 (18.4)	84 (8.4)	1.57 (1.00-2.47)	0.05	2.22 (1.29-3.80)	0.004
Non-smoker	374 (74.9)	902 (90.7)	1		1	
Cage score (Cigarette)						
Dependent (Score 1-4)	28 (2800.0)	64 (914.3)	7.73 (0.15-400.92)	0.31		
Not dependent	1 (100.0)	7 (100.0)	1			
FTND score						
Score > 6 (High)	3 (0.6)	2 (0.2)	0.58 (0.06-5.26)	0.63		
Score < 6 (Low)	26 (5.2)	69 (6.9)	0.56 (0.31-1.01)	0.06		
Ex-smoker	98 (19.6)	23 (2.3)	5.70 (3.11-10.45)	<0.01		
Non-smoker	374 (74.7)	902 (90.6)	1			
Physical activity						
Inadequate	452 (90.0)	860 (86.3)	2.86 (1.82-4.48)	<0.01	3.09 (1.88-5.07)	<0.001
Adequate	50 (10.0)	137 (13.7)	1		1	
Sweet food						
Frequently	242 (48.2)	567 (57.0)	0.92 (0.69-1.23)	0.58		
Rarely	260 (51.8)	428 (43.0)	1			
Fat food						
Frequently	333 (66.9)	643 (64.9)	1.12 (0.83-1.53)	0.46		
Rarely	165 (33.1)	347 (35.1)	1			
Salty food						
Frequently	298 (59.4)	502 (50.4)	1.68 (1.25-2.27)	0.001	1.92 (1.38-2.67)	<0.001
Rarely	204 (40.6)	495 (49.6)	1		1	

## Results

*Demographic characteristics of the participants*

In this study, it was found that older participants were more likely to have colon cancer than the younger. Age group of 60 years and over had 12.22 times (95% CI = 5.20-28.69) higher chance for developing colon cancer. Age group of 40-59 years had 8.21 times (95% CI = 6.24-10.80) chance for developing colon cancer. Other socioeconomic factors found to be associated with colon cancer were education, incomes, occupation, etc. 

Participants with other chronic non-communicable diseases had higher chance for having colon cancer, especially those with family history of colon cancer. These participants had 312 times higher chance for being diagnosed with colon cancer (95% CI = 190.54-513.63) (Table 1).

The results of the study showed that the socioeconomic factors of age, occupation, and incomes were significantly associated with risk of colon cancer. 

*Comparison of physiological factors between two groups using univariate analysis*

Low BMI, high systolic blood pressure, high FBS, low HDL and, high triglyceride were associated with risk of developing colon cancer. There was no association between other physiological factors and colon cancer (Table 2).

*The multiple variables relationship between physiological factors and colon cancer risk*

After adjusting the influence of socioeconomic status, it was found that low BMI, high systolic blood pressure and low HDL were associated with colon cancer risk. There was no association between other physiological factors and colon cancer risk. Participants with BMI < 18.5 had 6.20 times higher chance (95% CI = 3.56-10.81) for developing this cancer compared with those with normal BMI. Participants with abnormal systolic pressure (> 160 mmHg) had 5.20 times higher chance for developing colon cancer (95% CI = 1.02-26.56) compared to those with normal systolic pressure (Table 3).

Using multivariate analysis, it was found that the status of physiological factors could predict the likelihood of developing colon cancer. For instance, Participants with low BMI (<18.5) had OR of 5.26 (95% CI = 1.10-25.14) compared to those with normal BMI (18.5-24.9). Participants with abnormal systolic blood pressure (> 160 mmHg) or more found to have 30.48 times higher chance of developing this cancer (95% CI = 1.85-502.85) compared to those with normal systolic pressure. Participants with HDL less than 40 mg / dL had 8.80 times higher chance for having this cancer (95% CI = 2.62-29.25) (Table 3). 

*Association between behavioral factors and colon cancer risk*

*Association between behavior factors and colon cancer risk based on univariate analysis*

Smoking, low physical activity, and eating sweet, fatty, and salty food were found to be associated with colon cancer risk (Table 4).

*Exploring the association between behavior factors and colon cancer risk using multivariate analysis*

After adjusting the socioeconomic status, no association was observed between alcohol consumption and AUDIT score and colon cancer risk, while an association was found between smoking and colon cancer risk. Participants who smoked had higher chance for developing colon cancer revealing dose-response relationship (less than 20 pack a year, OR =1.57 times; 95% CI = 1.00-2.47 and more than 20 pack a year, OR = 3.00 times; 95% CI = 1.19-7.55). In addition, it was found that participants with inadequate physical activity had higher chance for developing colon cancer (OR = 2.86, 95% CI = 1.82-4.48) compared to those who had adequate physical activity. Participants who regularly ate salty foods had higher chance of developing colon cancer (OR = 1.68, 95% CI = 1.25-2.27) (Table 5).

Multivariate analysis results showed that smoking, inadequate physical activity, and eating salty foods were associated with colon cancer risk. Those who smoked more than 20 packs a years had 3.25 fold chance of developing bowel cancer (OR = 3.25, 95% CI = 1.17-9.03). Those with inadequate physical activity had 3.09 times of having colon disease (OR = 3.09, 95% CI = 1.88-5.07). Participants who regularly ate salty foods had also higher chance of developing colon cancer (OR = 1.92, 95% CI = 1.38-2.67) (Table 5).

## Discussion

Many factors are associated with abnormal division and growth of colonic epithelial cells. The abnormal growth will eventually progress to cancer. Patients usually come to hospital with symptoms of colonic obstruction or bleeding. Diagnostic gold standard for bowel cancer is tissue diagnosis. The colon cancer is divided into 3 types based on its etiology, namely sporadic colon cancer, familial colon cancer, and hereditary colon cancer. In patients with sporadic colon cancer, there is no history of colon cancer in the family. About 70-85% of all colon cancer patients are in this group. More than 80% of the patients aged more than 50 years-old. However, patients with familial colon cancer always have a history of colon cancer in their family member. In this type, abnormal growth is due to genetic factors. This group comprises 20-25 % of all colon cancer patients (Claes et al., 2011). The third type is found in about 5-10 % of all colorectal cancer patients. whose inheritance are familial polyposis (FAP), hereditary nonpolyposis syndromes (HNPCC) (Knudsen et al., 2003). Neverthelse, there are modifiable and unmodifiable factors contributing to development of this cancer. 

Modifiable risks factors can be obesity, smoking, heavy alcoholic consumption, and lack of physical activity. Overweight and obesity can cause changes in the body leading to cancer, such as increase in levels of certain hormones and inflammation. There are 13 types of cancer associated with overweight and obesity, including colon cancer (Massetti et al., 2017). Tobacco contains at least 70 carcinogenic chemicals getting into bloodstream and damaging DNA. Damaged DNA can lead to abnormal cells growth and cancer. Alcohol breaks down into acetaldehyde. Acetaldehyde can damage DNA. The damaged DNA can lead to cancer development (Humans, 2010). Inadequate physical activity is associated with many cancers in human. Understanding the biological mechanisms underlying the associations between physical activity and cancer will provide needed insights for reducing cancer risk. For colon cancer, the major hypothesis is that physical activity lowers fecal bile acid concentrations and decreases gastrointestinal transit time (Clague and Bernstein, 2012; Wolin et al., 2009).

The results of this study on Thai patients with colon cancer revealed that age and socioeconmic factors were associated with colon cancer risk. In this study, family history of colon cancer was significantly associated with colon cancer risk. It was also found that physiological abnormalities such as high systolic blood pressure, dyslipidemia (low HDL), and low BMI were associated with colon cancer risk. However, our results may not be reliable due to missing data. The missing laboratory data in both group were due to nature of our study. 

Behavioral factors, including smoking, inadequate physical exercise, and salty food consumption, were associated with colon cancer risk. We found no association between obesity and alcohol consumption and colon cancer. The results suggested that genetic factors may have higher association with colon cancer risk among Thai patients than behavioral factors. 

## References

[B1] Bye WA, Nguyen TM, Parker CE, Jairath V, East JE (2017). Strategies for detecting colon cancer in patients with inflammatory bowel disease. Cochrane Database Syst Rev.

[B2] Chamroonsawasdi K, Chottanapund S, Tunyasitthisundhorn P (2017). Development and validation of a questionnaire to assess knowledge, threat and coping appraisal, and intention to practice healthy behaviors related to non-communicable diseases in the Thai population. Behav Sci (Basel).

[B3] Claes K, Dahan K, Tejpar S (2011). The genetics of familial adenomatous polyposis (FAP) and MutYH-associated polyposis (MAP). Acta Gastroenterol Belg.

[B4] Clague J, Bernstein L (2012). Physical activity and cancer. Curr Oncol Rep.

[B5] Conigrave KM, Hall WD, Saunders JB (1995). The AUDIT questionnaire: choosing a cut-off score Alcohol Use Disorder Identification Test. Addiction.

[B6] Demirbas H (2015). Substance and alcohol use in young adults in Turkey as indicated by the CAGE questionnaire and drinking frequency. Noro Psikiyatr Ars.

[B7] Ebbert JO, Patten CA, Schroeder DR (2006). The fagerstrom test for Nicotine Dependence-Smokeless Tobacco (FTND-ST). Addict Behav.

[B8] Faivre J (1994). Colon cancer Epidemiology pathological anatomy diagnosis course treatment and prevention. Rev Prat.

[B9] Humans IWG. o. t. E. o. C. R. t. (2010). Alcohol consumption and ethyl carbamate. IARC Monogr Eval Carcinog Risks Hum.

[B10] Kessaram T, McKenzie J, Girin N (2015). Noncommunicable diseases and risk factors in adult populations of several Pacific Islands: results from the WHO STEPwise approach to surveillance. Aust N Z J Public Health.

[B11] Khuhaprema T, Srivatanakul P (2008). Colon and rectum cancer in Thailand: an overview. Jpn J Clin Oncol.

[B12] Knudsen AL, Bisgaard ML, Bulow S (2003). Attenuated familial adenomatous polyposis (AFAP) A review of the literature. Fam Cancer.

[B13] Lepage C, Hamza S, Faivre J (2010). Epidemiology and screening of colon cancer. Rev Prat.

[B14] Lerdkiattikorn P, Chaikledkaew U, Lausoontornsiri W (2015). Cost-utility analysis of adjuvant chemotherapy in patients with stage III colon cancer in Thailand. Expert Rev Pharmacoecon Outcomes Res.

[B15] Massetti GM, Dietz WH, Richardson LC (2017). Excessive weight gain, obesity, and cancer: Opportunities for Clinical InterventionAssociation Among Excessive Weight Gain, Obesity, and CancerAssociation Among Excessive Weight Gain, Obesity, and Cancer. JAMA.

[B16] Nordqvist C, Johansson K, Bendtsen P (2004). Routine screening for risky alcohol consumption at an emergency department using the AUDIT-C questionnaire. Drug Alcohol Depend.

[B17] Slattery ML, West DW, Robison LM (1990). Tobacco, alcohol, coffee, and caffeine as risk factors for colon cancer in a low-risk population. Epidemiology.

[B18] Uysal MA, Kadakal F, Karsidag C (2004). Fagerstrom test for nicotine dependence: reliability in a Turkish sample and factor analysis. Tuberk Toraks.

[B19] Wheat CL, Clark-Snustad K, Devine B (2016). Worldwide Incidence of Colorectal Cancer, Leukemia, and Lymphoma in Inflammatory Bowel Disease: An Updated Systematic Review and Meta-Analysis. Gastroenterol Res Pract.

[B21] WHO (2018). Noncommunicable diseases country profile 2018.

[B22] Williams N (2014). The CAGE questionnaire. Occup Med (Lond).

[B23] Wolin KY, Yan Y, Colditz GA, Lee IM (2009). Physical activity and colon cancer prevention: a meta-analysis. Br J Cancer.

